# Optimisation of a One-Step Reusable Immuno-Affinity Purification Method for the Analysis and Detection of Fumonisin Mycotoxins in Foods and Feeds

**DOI:** 10.3390/toxins17110538

**Published:** 2025-10-30

**Authors:** Christian Kosisochukwu Anumudu

**Affiliations:** 1 School of Chemical Engineering, University of Birmingham, Birmingham B15 2TT, UK; cka329@student.bham.ac.uk; 2 Institute for Global Food Security, Queens University Belfast, Belfast BT9 5DL, UK; 3 Department of Microbiology, Federal University Otuoke, Otuoke 562103, Bayelsa State, Nigeria

**Keywords:** Ultra-high performance liquid chromatography, Fumonisin, Tandem mass spectrometry, Immuno-affinity purification, Maize, Mycotoxicosis

## Abstract

Fumonisins are among the most prevalent mycotoxins in maize and maize-based products, posing significant food safety and public health risks due to their hepatotoxic, nephrotoxic, and potential carcinogenic effects. Given the strict regulatory limits set by the European Commission and Codex Alimentarius, the development of reliable, sensitive, and matrix–robust analytical methods remain a priority for routine monitoring in both food and feed systems. In this study, a reusable immuno-affinity purification methodology for the quantitative determination of fumonisin mycotoxins (FB1, FB2 and FB3) in foods and feeds (maize matrix) was developed. A single extraction protocol using 2% formic acid in water was employed, followed by cleanup with an immuno-affinity purification column and toxin elution by methanol/PBS (1:1, *v*/*v*). Detection and quantification of the mycotoxins was achieved by a normal phase ultra-high performance liquid chromatography coupled with electrospray ionisation triple quadrupole mass spectrometry (UHPLC/ESI-MS/MS). The chromatographic mobile phase utilised was a linear gradient of methanol/water containing 0.1% formic acid. The developed method has a limit of detection of 2.5 ng/g and a limit of quantification of 5 ng/g, all well below the European commission’s guidance values of 1000 ng/g for corn destined for human consumption and 800 ng/g for maize-based breakfast cereals and snacks. While the recovery rates of the method in this study ranged from 65–70% for the three fumonisin analogues in solutions, when tested in maize matrix, recoveries were markedly lower (~30%) due to pronounced matrix suppression. Good repeatability (standard deviation <10%) was achieved for all the fumonisin analogues. The developed method, although quick and effective in solvent systems, suffered limitations to its practical usage due to matrix suppression of the extracts derived from the immuno-affinity purification column, thus significantly reducing the application of the method in measuring fumonisin mycotoxins in food and feed samples. Overall, the method was effective in quantification of fumonisin mycotoxins in solvent solutions but not in food and feed matrices, thus necessitating further optimisation for practical usage. The performance of the developed method was compared to a commercial lateral flow immunochromatographic assay which proved to be better than the developed method in the quantification of toxins in food matrices, as the commercial lateral flow immunochromatographic assay outperformed the developed method in maize matrices. These findings highlight the need for matrix-based validation and further refinement of antibody stability to ensure robust application in regulatory monitoring of fumonisins using immunoaffinity purification methods.

## 1. Introduction

**Figure 1:**
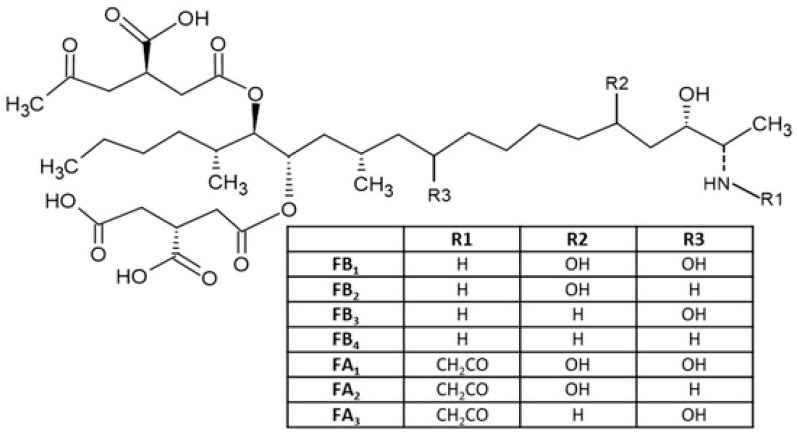
Basic structure of different fumonisins [5].

The fumonisins are major mycotoxins produced by a vast number of fungi, including *Fusarium verticillioides* and *Fusarium proliferatum* [1]. They are common contaminants of corn and maize-based or maize-derived products worldwide, which is reportedly the most commonly affected host plant [2]. The fumonisins are classified into four major groups (A, B, C and P). Of these, the group B fumonisin analogues are the most abundant. The group B-series are further divided into FB1, FB2 and FB3, of which Fumonisin B1 (FB1) accounts for about 70% of all fumonisin contaminations in maize and maize based products [3]. Of the fumonisins, FB1 has the highest toxicity and is classified by the International Agency for Research on Cancer (IARC) as a possible (class 2B) carcinogen [4]. The basic structure of fumonisin mycotoxin is shown in Figure 1.

Ingestion of foods or feeds contaminated with the fumonisins can lead to various human and animal diseases, including neural tube defects and esophageal cancer in humans [6], porcine pulmonary edema in pigs [7], and equine leukoencephalomalacia in horses [8]. Because of the health concerns associated with the consumption of the fumonisins, there have been several recommendations and regulations that limit the amount of fumonisins present in foods and feeds destined for human and animal consumption [9,10]. Within the European Union, Commission Regulation (EU) 2023/915, which set maximum levels of contaminants in foods, stipulates a maximum fumonisin level of 1000 ng/g for corn destined for direct human consumption, 200 μg/kg for corn-based baby foods and 800 ng/g for corn-based snacks and breakfast cereals [11].

Numerous analytical methodologies have been developed for the detection of the fumonisins in foods and feeds. These include immunological methods such as Enzyme Linked Immuno-Sorbent Assays (ELISAs) and lateral flow devices (LFD) [12], as well as chromatographic techniques such as thin-layer chromatography (TLC), gas chromatography–mass spectrometry (GC/MS) and various forms of liquid chromatography coupled with different detectors, including mass spectrometry (LC/MS), high resolution mass spectrometry (LC-HRMS) and fluorescence detection (LC/FLD) [13]. Of these, the most frequently utilised analytical methodology is high performance liquid chromatography coupled to a fluorescence detector (HPLC-FLD) [3]. However, detection of the fumonisins using this methodology requires prior derivation, which can cause false results due to co-elution of interfering compounds or retention time shifts [14]. The use of liquid chromatography coupled to tandem mass spectrometry (LC-MS/MS), employing a triple quadrupole (QqQ) analyser in multiple reaction monitoring (MRM) mode, has emerged as a preferred modern method, as it allows for the detection of underivatised fumonisins at trace levels in complex matrices [14,15].

Various extraction and purification methods have been developed for the clean-up of samples prior to HPLC-MS detection. The most utilised clean up methodologies involve extraction with organic solvents such as acetonitrile alone or in association with water acidified with different percentages of formic or acetic acid and clean-up achieved by strong anion exchange (SAX) columns and Solid-Phase Extraction (SPE) [16]. These methods are relatively tedious, requiring extensive sample manipulations, and produce much toxic waste. With the trend towards more rapid and automated procedures due to increasing number of test samples, other methodologies are being employed. Generally, these are QuEChERs (Quick, Easy, Cheap, Effective, Rugged and Safe) and involve extraction by use of a mixture of water acidified with formic acid and acetonitrile, with purification achieved by the use of magnesium sulphate (MgSO_4_) and sodium chloride (NaCl) (for salting out various matrix components) [17], as well as liquid–liquid extraction (LLE) and pressurised liquid extraction (PLE) [18]. Despite broad usage, these extraction and clean-up procedures have limitations. Conventional SAX and SPE methodologies are labor-intensive, time-consuming, and produce large volumes of toxic organic solvents that raise environmental and safety concerns [19]. While QuEChERS has optimised the procedure, it is not wholly efficient in removing matrix interferences [20]. Similarly, while LLE and PLE reduce solvent consumption compared to conventional techniques, they also require specialised equipment and suffer from limited reproducibility concerns when applied to a range of sample matrices [21]. These drawbacks highlight the ongoing need for more efficient, high-throughput, and less environmentally degrading techniques for the detection of fumonisins.

Research is shifting towards the use of immuno-affinity columns (IAC) for the clean-up and pre-concentration of various mycotoxins [22]. In IAC purification, columns are prepared by coupling antibodies that are specific for a particular mycotoxin to a specialised biosupport packed into a column/cartridge. The sample to be purified is passed in solution through the IAC column. Mycotoxins in the sample extract bind to the antibodies immobilised in the column, while impurities are removed by an aqueous rinse step. Trapped mycotoxins are eluted from the column using elution solvents such as methanol. Immuno-affinity columns offer high selectivity and specificity for toxins because the antibodies bind only to the toxin of interest. Also, this methodology reduces the production of hazardous wastes, increases the speed of purification and can detect low concentrations of the analytes of interest [23]. Importantly, the performance of immuno-affinity columns is largely dependent on the biosupport and antibody properties [24]. The usual biosupports employed are agarose gels, silica microbeads and synthetic polymers that are used to provide mechanical strength and have large surface areas for affixing the antibodies [23]. The choice of biosupport decides not only binding efficiency but also flow rates, column stability, as well as the possibility of reusing them. Likewise, the kind of antibody employed is also a deciding factor for selectivity and specificity. Polyclonal antibodies, while relatively inexpensive and of broad reactivity, suffer from batch-to-batch variability. Monoclonal antibodies, which have uniform and highly specific binding to a single epitope, are therefore preferred for consistent toxin elution [25]. However, some of the disadvantages of immuno-affinity columns are that at high concentrations of analytes, the capacity of the IAC may be exceeded, as there are limited antigen binding sites on the antibodies [26]. Furthermore, most IAC columns can only be used once, thus increasing the cost of analysis by this methodology. Also, compounds with similar molecular weight or structure as the analyte of interest may competitively bind to the antibodies, resulting in an underestimation of analytes present [23].

This study was undertaken to develop a method for the quantification of fumonisin mycotoxins (FB1, FB2 and FB3) in feeds using a reusable immuno-affinity purification column (IAC) for extraction and purification of the mycotoxins prior to analysis by ultra-high performance liquid chromatography tandem mass spectrometry (UHPLC-MS/MS). Although LC–MS/MS dilute-and-shoot methods have been widely used for mycotoxin determination and can achieve acceptable recoveries under EU regulations, they often encounter matrix effects and reduced sensitivity in complex or low-contamination matrices such as corn-based baby foods and processed maize feeds. The rationale of this study was therefore to develop a cost-efficient, one-step immuno-affinity purification method that minimizes matrix interferences while enabling accurate detection of fumonisins at trace concentrations. The limit of detection and quantification of the method was validated for quantification of fumonisin mycotoxins using toxin standards dissolved in PBS buffer systems. Performance of the developed purification method was compared with a commercial lateral flow immunochromatographic assay with the aim of benchmarking the developed method against a widely used rapid screening tool. It was also investigated whether the presence of masked fumonisins and compounds within the sample matrixes may interfere with the functionality of the immuno-affinity purification column by competitively binding to the immobilised antibodies within the column.

## 2. Results and Discussion

### 2.1. Extraction and Immuno-Affinity Clean-Up Optimisation

Many of the previously published studies show that the most effective and widely utilised solvent for multi-mycotoxin extraction is an 80:20 (*v*/*v*) mixture of acetonitrile and water containing acetic or formic acid [27,28]. This technique has been widely adopted because protein-mycotoxin interactions can be efficiently disrupted by acetonitrile, which enhances toxin recovery across diverse food matrices [29]. However, fumonisins differ from many other mycotoxins in that they are relatively hydrophilic, dissolve completely in polar organic solvents such as methanol and acetonitrile:water (1:1) mixtures, and do not exhibit native fluorescence [30]. While these unique properties are advantageous for solubility, they raise concerns that commonly used solvent systems could compromise the binding efficiency of antibodies in immuno-affinity purification columns. Hence for this study, the efficacy of the antibodies in the immuno-affinity columns to trap analytical standards of fumonisins dissolved in various solutions including; PBS, H_2_O acidified with 2% formic acid, acetonitrile (MeCN) and MeCN: H_2_O (1:1, *v*/*v*) was investigated.

Of all the extraction solvents investigated, H_2_O acidified with 2% formic acid gave the highest recovery (measured as peak areas). In contrast, pure acetonitrile suppressed the binding activity of antibodies within the immuno-affinity purification (IAC) column. This agrees with previous findings that acidic conditions decrease the binding efficiency of antibodies immobilised in the IAC column [31,32], but are however necessary for the effective extraction of fumonisin mycotoxins, highlighting the need for careful optimisation of the choice and proportion of organic solvents [33]. Hence, our samples were extracted with H_2_O acidified with 2% formic acid and the resulting extracts are subsequently diluted (1:4) with PBS before passing through the IAC columns, thereby lowering the proportion of the organic solvent to levels compatible with antibody functionality. This two-step approach balances efficient analyte release from the food matrix with optimal antibody–antigen interactions and has also been reported in other recent immune-affinity studies [23,34,35].

After loading the column, a significant challenge was to elute the toxins without damaging the antibodies, while ensuring optimum recovery. Different solvents were investigated for elution of toxins. It was found that 100% methanol (MeOH) was very effective in eluting bound toxins, but the column loses its functionality rapidly. Although glycine buffer has been recommended as a mild elution system to preserve column reusability [36], in this study it was ineffective even at higher concentrations of 0.2M at a pH of 2.5. Elution was found to be stable and repeatable when MeOH: PBS (1:1, *v*/*v*) was utilised as the elution solvent. This behaviour could be due to two reasons. First, the addition of an aqueous buffer component (PBS) reduces the denaturing effect of pure organic solvent on immobilised antibodies, which helps to preserve antibody tertiary structure and binding sites during the elution step [37]. For this reason, several immunoaffinity protocols and reviews have recommended aqueous–organic mixtures over ordinary organic solvents [23,38]. Since fumonisins are relatively polar, mixed aqueous–organic eluents could improve their solubility and desorption from antibody binding sites in comparison with highly organic eluents [39,40]. Recoveries of the different elution solvents are summarised in Table 1.

### 2.2. Performance Characteristics of Developed Method

Blank corn samples fortified/spiked with different levels of FB1, FB2 and FB3 were utilised to determine the recovery rate, linearity, limits of detection (LOD) and limit of quantification (LOQ) of the developed method.

For the measurement of LOD and LOQ, certified blank corn matrices were spiked/fortified with decreasing concentrations of the fumonisin mycotoxins. An unfortified/unspiked corn sample was also analysed to measure the baseline noise ratio. Limit of detection (LOD) was defined as the lowest concentration of the mycotoxins, which yielded a signal that is three times higher than the noise ratio obtained from the blank corn samples. All spiked and unspiked samples were measured in triplicates to ascertain the recovery rates and linearity of the developed method. The limit of quantification (LOQ) was defined as the lowest concentration of toxins from which the quantifier ion had a signal-to-noise ratio greater than 10 and which showed sufficient accuracy (<25% deviation) in both intra-day and inter-day assays.

The developed method achieved an LOD of 2.5 ng/g and LOQ of 5 ng/g, which is well below the current guidance values set by Commission Regulation (EU) 2023/915, which stipulates a maximum fumonisin level of 1000 ng/g for corn destined for direct human consumption, 200 ng/g for corn-based baby foods and 800 ng/g for corn-based snacks and breakfast cereals [11]. Compared to contemporary analytical methods, our LOD of 2.5 ng/g and LOQ of 5 ng/g are highly competitive. For instance, a recent LC-MS/MS validation study by Masquelier et al. [41] reported LODs of 50 ng/g and LOQs of 100 ng/g for FB1, FB2 and FB3, respectively, in maize. More broadly, multi-toxin LC-MS/MS techniques typically report LODs/LOQs in the range of 10–100 ng/g [29], further highlighting the strength of our approach.

### 2.3. Recovery, Repeatability and Inter-Day Reproducibility of Developed Method

Spiked corn matrix and PBS buffer samples were analysed at three concentration levels (50, 80 and 150 ng/mL). As shown in Table 2, the recovery rates obtained were constant within each of the corn matrices and PBS solvents but hugely differed between the two. With this method, the mean recovery rate in intra-day studies obtained from the spiked PBS solvents was about 62% (FB1), 68% (FB2) and 68% (FB3). The recovery obtained from the spiked corn matrixes was very low (about 30%). The huge difference between PBS recoveries (~62–70%) and corn matrix recoveries (~30–33%) can be attributed to a pronounced matrix-dependent suppression effect indicative of either reduced analyte binding to the immunoaffinity column, elution problems or impaired ionisation efficiency in the LC-MS/MS detection step. This is consistent with recent findings showing that co-extractives such as proteins, lipids, starches, pigments and bound or masked fumonisin forms can suppress LC–MS signals or interfere with antibody–antigen binding in IAC columns [42]. Statistically, the low recoveries observed in the maize matrix fall far below the minimum acceptable limit of 70% typically recommended by regulatory validation guidelines for quantitative residue methods.

It is important to note that apparent recoveries in solvent systems tend to exaggerate method performance because they do not account for matrix interferences in the real food or feed matrices. Complex matrices can lead to adsorption, degradation or ion suppression/enhancement and may result in significant deviations of the recoveries realised in solvent-only systems [43]. Hence, there is need for recovery studies in real matrices rather than only relying on apparent recoveries from solvent-based systems.

The repeatability of the whole method in intra-day studies was very good for both the corn matrix and spiked PBS buffer solution. Intra-day variation coefficients (calculated as standard deviation × 100/Mean; presented in Table 2) obtained from PBS spiked buffer solutions of FB1, FB2 and FB3 are as follows: 0.6- 13.1%, 3.5–9.4% and 4.1–8.9%, respectively. All relative standard deviation (RSD) values calculated for PBS samples fall within the commonly accepted threshold of ≤15% for repeatability. At the 50 ng/g level, FB1 exhibited the lowest intra-day RSD (0.65%), but at 80 ng/g exhibited the highest (12.5%).

The developed purification method was very reproducible in inter-day studies, as presented in Table 3. Inter-day reproducibility studies were performed by measuring spiked samples (corn matrix and PBS buffer solutions) on two separate days. The inter-day variation coefficients obtained for the three fumonisin analogues (In PBS buffer solutions) ranged from 2.3–13.8%, irrespective of the fortification level. These coefficients of variation (CVs) for inter-day reproducibility are consistent across concentrations and analytes, with all values remaining below 15%, indicating low between-day analytical variation under controlled conditions.

These precision metrics indicate that the method is precise (repeatable and reproducible) when matrix and instrument conditions are stable. However, while precision is acceptable, the recoveries in maize are well below those reported in validated IAC–LC–MS/MS protocols. Recent studies typically achieved mean recoveries within the acceptable range of 74–108%, with RSDs ranges of 12.0 to 29.8% in maize [41,44], indicating that recoveries of ~30% in this study are substantially lower than contemporary benchmarks. No statistical outliers were observed within replicate sets (*n* = 3) at each concentration level, and the standard deviations remained within ±9% in all cases, supporting consistent analytical performance under repeat conditions.

The very low recoveries of 30–33% obtained from the spiked corn matrix contrast with results obtained from a previous study by Guan et al. [45], which recorded recoveries of ~85% for the fumonisins. For corn matrix samples, inter-day recovery standard deviations ranged from 0.1 to 2.6%, corresponding to RSDs between 0.3% and 8.2%. In contrast, PBS-spiked samples showed higher recovery means (64.5–70.4%), with inter-day SDs ranging from 1.6 to 9.7%, and corresponding RSDs between 2.5% and 13.9%, calculated using (SD/mean) × 100. The low recoveries obtained in this study may be because of matrix interference/suppression of antibody activity within the immuno-affinity purification column. Other possible reasons could be due to the incomplete release of fumonisins from the matrix during extraction, the presence of matrix components that competitively bind or block antibody binding sites in the IAC, or the strong ion suppression in the LC–MS stage despite IAC cleanup. Similar conclusions were drawn by Damiani and colleagues [46], who demonstrated that sample particle size and solvent composition strongly influence fumonisin extractability.

This low recovery significantly reduces the potential of this method in its current form as a quantitative analytical tool for measurement of fumonisins in foods and feeds. This is because the recovery rate does not meet up to the criteria stipulated by Commission Implementing Regulation (EU) 2023/2782 for acceptable mycotoxin analyses, which requires recoveries between 70 and 120% with a relative standard deviation ≤ 25% [47]. From a food safety risk assessment perspective, such low recoveries can lead to systematic underestimation of fumonisin concentrations, potentially resulting in false compliance with regulatory limits and an underappreciation of true exposure risks to consumers. Furthermore, this analytical bias may compromise the reliability of contamination data used for dietary exposure estimation and policy decisions. However, the huge discrepancy in results between the PBS and maize matrix highlights the extraction and cleanup stages as the primary bottlenecks in our method. The variance between mean recoveries in PBS and corn matrices exceeded 35% points for all analytes, and this matrix effect was consistent across all three concentration levels, indicating statistically significant suppression likely occurring upstream of the detection phase. This suggests that optimisation of parameters like the re-examination of extraction efficiency using alternative solvents such as MeCN:H_2_O (80:20) with acidification might yield significant improvements to reach standard benchmarks [48].

**Figure 2:**
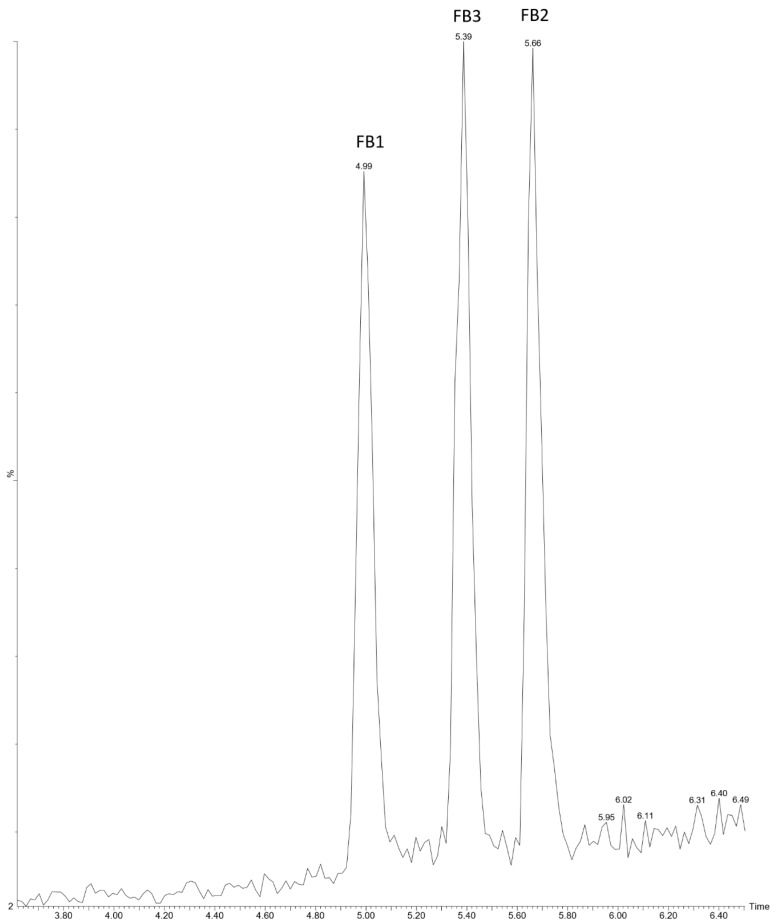
UHPLC-MS/MS chromatogram of fumonisin showing peaks for FB_1_ (4.99 min), FB_3_ (5.39 min), and FB_2_ (5.66 min).

The chromatogram in Figure 2 depicts the separation and detection of the three major fumonisin analogues (FB1, FB2, FB3) using the optimised UHPLC-MS/MS method. The obtained retention times were 4.99 min for FB1, 5.66 min for FB2, and 5.39 min for FB3, which indicates good chromatographic resolution in a relatively short run time. This rapid elution is in line with the performance expectations of UHPLC approaches, providing increased sensitivity and resolution compared to standard HPLC approaches [49,50]. The clear resolution between the peaks indicates the success of the column and mobile phase conditions to minimise co-elution, particularly when handling complex food matrices with high potential for interference. Interestingly, the separation of FB1 (4.99 min) and FB3 (5.39 min) also validates the success of the method in simultaneous quantification of fumonisin analogues. In comparison with other studies, the retention times observed here are consistent with those generated for UHPLC and LC-MS/MS methods. For example, Badr et al. [51] reported retention times of 5.0–6.0 min for FB1 and FB2 under similar chromatographic conditions, and Masquelier et al. [41] reported a retention time of between 10.2 to 11.1. This concurrence indicates the robustness of the current method. Furthermore, studies have reported that complex cereal matrices can cause substantial peak broadening or retention time shifts for fumonisins during UHPLC–MS/MS analysis, leading to inaccurate quantification if not properly controlled. For example, Fabregat-Cabello et al. [52] demonstrated that under non-optimised conditions, notable retention instability and signal suppression occurred for FB_1_ and FB_2_ in maize extracts. In contrast, the present procedure had replicable retention times, which suggests less variability induced by the matrix, at least with the optimised conditions used. A desirable stability in this aspect is that peak overlap or shift can impact the accuracy of quantitation in routine mycotoxin monitoring. In practice, the short analysis time (<6 min) and the high resolution make the method highly suitable for high-throughput monitoring, which is an important prerequisite in regulatory testing where many food and feed samples must be analysed within a limited time.

### 2.4. Matrix Effect Studies

A study of matrix effect on the activity of the IAC column was undertaken by comparing calibration standards prepared in PBS to matrix-matched standards. From the results obtained as shown in Table 2 and Table 3, there is very significant matrix suppression (up to 70%) of the sample (corn) extracts derived from the immuno-affinity purification column in relation to the standards prepared in pure PBS solvents. This result was not anticipated because the immobilised antibodies within the column are expected to trap only the fumonisin toxins, and a subsequent wash step is expected to remove all matrix components. The large suppression observed is consistent with findings from recent studies that demonstrated that cereal matrices can substantially reduce apparent recoveries in IAC-LC–MS/MS workflows due to co-extractives and modified/masked mycotoxins that reduce effective analyte capture or produce ion suppression in the MS source [46,53,54]. Furthermore, a possible explanation for this matrix suppression is that there are compounds within the matrix that have similar chemical structure and/or molecular weight as the fumonisins that competitively bind to the antibodies, preventing the complete trapping of fumonisin mycotoxins by the antibodies; these substances are not being detected by mass spectrometry, possibly because the mass spectrometer was operated in the selective ion monitoring mode and the compounds do not yield daughter ions similar to the fumonisins. The plausibility of this competitive-binding hypothesis has been reported in several studies. For example, Zhang et al. [55] developed a direct competitive ELISA based on magnetic beads (MBs-dcELISA) that could simultaneously detect total aflatoxins (AFs, AFB1, AFB2, AFG1 and AFG2). To overcome false-positive and false-negative results that are caused by high or low cross-reactivity of antibodies, they generated a new broad-specific monoclonal antibody (5H3) with uniform affinity. They used magnetic beads as the immobilisation phase, instead of conventional microplates, to further improve the sensitivity of the assay. They found that this approach effectively reduced matrix interference and yielded consistent detection across all four major aflatoxins, with LOD of 0.21 ng/g for the total Afs, recoveries between 74.5 to 96.5% and good agreement with HPLC-MS/MS. Their findings demonstrate that the optimisation of antibody specificity and assay design can mitigate the same competitive-binding challenges observed in fumonisin analysis, which may either arise from cross-reactive matrix components or modified toxin derivatives. To investigate if there are other compounds being trapped by the antibodies in the immunoaffinity column, the mass spectrometer was set to the full scan mode. With the theory that during the full scan, if peak chromatograms other than the fumonisins is observed, this will indicate the presence of other compounds in the matrix competitively binding the antibodies in the IAC column. However, no other peak was observed in the full scan mode. This negative result is not definitive. First, the instrument used is a low-resolution mass spectrometer and full-scan sensitivity/resolving power may be insufficient to reveal low-abundance co-eluting compounds or high-mass adducts; however, modern high-resolution MS (HRMS) instruments can detect modified fumonisins and conjugates missed by low-resolution scans [56]. Second, some modified forms are only revealed after targeted hydrolysis or specific MS/MS methods; absence of peaks in an untargeted low-resolution scan therefore does not exclude competitive trapping or masked species [56]. Another explanation may be that the fumonisin mycotoxins within the samples react with the matrix, yielding fumonisin conjugates/masked fumonisins which are not able to bind to the antibody. This is supported by several studies that have demonstrated that maize contains modified/‘masked’ fumonisin forms and matrix-bound/conjugated species that can escape routine analysis unless hydrolysed or specifically targeted [56,57].

To investigate the presence of masked fumonisins, alkaline hydrolysis of the corn samples was undertaken, making use of potassium hydroxide. Upon hydrolysis of the sample, the following transitions of the hydrolysed fumonisin ions were obtained; 406.5 → 370.5 and 406.5 → 388.5 for HFB1, 390.5 → 336.4 and 390.5 → 372.5 for HFB2 and HFB3. These transitions are similar to hydrolysed fumonisin product ions reported in the study by Generotti et al. [58] following alkaline hydrolysis which allowed for the estimation of total (free + bound) fumonisins. Importantly, the quantification of hydrolysed fumonisin requires appropriate standards or isotope-labelled internal standards to ensure accurate recovery correction and quantitation, because without hydrolysed standards the results can only be treated qualitatively or semi-quantitatively [59,60]. Due to a lack of hydrolysed fumonisin standard solutions and time constraints, the amount of masked fumonisins within the samples could not be quantified and compared to the free fumonisin toxins to investigate their presence in relation to the free fumonisins. Future studies can integrate other approaches that have been successfully applied in recent studies, such as the acquisition or synthesis of hydrolysed fumonisin standards [61], the use of HRMS to screen for unknown conjugates [62] and isotope-dilution quantification to correct for matrix effects and losses during hydrolysis [53].

### 2.5. Performance of the Lateral Flow Immunochromatographic Assay

Recovery rates of the fumonisin mycotoxins from spiked corn matrix and PBS buffer solutions were measured in duplicates at three levels of fortification (400 ng/g, 500 ng/g and 750 ng/g) using a lateral flow immunochromatographic device (Reveal Q+ for fumonisin). The recoveries are presented in Table 4. The recovery rate of the lateral flow device was constant for both toxins spiked in PBS and in corn matrix at about 70% for spiked corn matrix and 80% for toxins in PBS buffer.

The recovery rates obtained from measuring spiked PBS buffer solutions by the lateral flow device (78%) are comparable to those obtained from eluting spiked PBS buffer solutions from the IAC (68%). The relative difference in mean recovery between PBS (78.3%) and corn matrix (68.2%) is approximately 10.1 percentage points. However, for practical purposes of measuring toxin levels in food matrices, the results of the lateral flow device (68%) contrast with the recovery obtained from the immunoaffinity purification column (30%), which was only able to efficiently trap toxins in PBS buffer solutions and not in the corn matrix. This divergence is plausible considering that LFIAs capture analyte directly from a relatively crude extract and can be less sensitive to certain binding interferences that affect the chemistry of IAC capture. Conversely, IAC-LC-MS/MS offers superior selectivity but can suffer capacity competition or antibody inhibition when the composition of the extract is unfavourable. Furthermore, comparable patterns, such as LFIA giving reasonable screening recoveries while LC-MS/MS workflows reveal significant matrix suppression or binding losses, have been reported across mycotoxin classes, which demonstrate that screening and confirmatory platforms respond differently to matrix co-extractives [63,64]. The anti-fumonisin 5D2 antibody utilised in the development of the immuno-affinity column was supplied by Neogen Corporation and is similar to that used in the Reveal Q+ Neogen immunochromatographic assay. Hence, a comparable result was expected. The discrepancy is most rationally explained by format-specific antibody environment and matrix conditioning. To clarify, the comparison presented here is intended to illustrate relative recovery behaviour between LFIA and IAC under equivalent matrix conditions, rather than to suggest quantitative equivalence in analytical performance or calibration accuracy. Notably, identical (or similar) antibodies can behave differently across formats: unlike IAC that rely on static binding within a packed bed and are limited by solvent tolerance and binding capacity, LFIA use membrane-immobilised antibodies with flow-through kinetics [65,66]. The addition of surfactants and blockers in LFIA further reduces nonspecific binding and matrix interference, thereby improving assay performance [67]. Kit inserts also specify strict extraction solvents and dilution steps that condition the matrix to be “LFIA-friendly,” which may not map directly to IAC loading conditions. These format-specific chemistries explain why LFIA achieved ~68% in maize while the IAC column underperformed. To further illustrate the relative analytical performance, Table 5 compares the developed IAC–UHPLC–MS/MS method with the commercial LFIA and representative LC–MS/MS methods reported in literature.

### 2.6. Comparing the Developed Immuno-Affinity Purification Method to Commercial Lateral Flow Immunochromatographic Assay

Results obtained from the measurement of corn samples naturally contaminated with fumonisins (Triology laboratory certified reference materials) by both the immuno-affinity purification methodology and lateral flow immunochromatographic assay are presented in Table 6.

Comparing these results to the expected reference values, the lateral flow immunoassay outperformed the developed immuno-affinity purification method, which was not suitable for the quantification of fumonisin toxins in maize. It is observed that at higher concentrations of the mycotoxins (above 3000 ng/g), the lateral flow test losses its sensitivity and there is no differentiation of high toxin concentrations. This is characteristic of antibody-based lateral flow assays, with similar results reported by Molinelli et al. [70] who described a LFIA range up to 4000 ng/g and noted flattening at the upper end of the working range. Although this flattening effect does not severely distort qualitative detection, it may compromise quantitative reliability by producing compressed responses at high analyte concentrations. Hence, caution is required when interpreting LFIA results in samples exceeding the linear detection range. Notwithstanding this flattening of results at higher concentrations, the lateral flow immunochromatographic assay appears to be more rugged than the IAC in quantitative fumonisin mycotoxin measurement. Furthermore, the range of the observed mean recoveries for the lateral flow device (~54–79%) fall within the commonly reported ranges for screening LFIAs in grains, which typically provide rapid, semi-quantitative results suitable for on-site decision-making but not for definitive quantification [67,71]. While LFIA formats can either be colorimetric or fluorescent labels, optimised kits can reach higher recoveries and lower LODs, but their principal role remains screening rather than confirmation [72].

In theory, IAC coupled to UHPLC-MS/MS should provide more accurate quantification and higher specificity. However, the IAC method in this study consistently under-recovered fumonisins (9–57% across reference samples), indicating substantial matrix-related losses or binding interference. This implies that applying this IAC-UHPLC-MS/MS workflow without optimisation would underestimate actual fumonisin concentrations in maize samples. This is very critical because underreporting of mycotoxins has real consequences: it can lead to false assurances of safety, failure to withdraw contaminated food from the market and underestimation of population exposure in dietary risk assessments [73,74]. The toxicological characteristics of the specific toxin (acute, long-term toxicity, mutagenicity, teratogenicity and carcinogenicity), age and the degree of exposure all affect how seriously a person is affected by mycotoxin exposure [75]. Even at low levels of chronic exposure, these mycotoxins are known to pose different health risks [3]. Notably, exposure to several mycotoxins may produce distinct indications and symptoms compared to exposure to a single mycotoxin, which make them important food/feed contaminants [73]. Hence, poor recovery is not merely a technical fault, it carries substantial public health and regulatory risk.

## 3. Conclusions

The developed method for a one step purification of food matrixes by a reusable immuno-affinity purification column prior to measurement by liquid chromatography tandem mass spectrometry demonstrates potential for rapid fumonisin purification in clean solvents but suffers from significant matrix suppression in maize, currently limiting its practical applicability. Further optimisation is necessary to improve recovery rates and ensure reliable quantification in food and feed matrices. The performance of the developed method in the detection of toxins spiked in PBS solvents is satisfactory (65–70%), with limits of detection and quantifications (2.5 ng/g and 5 ng/g, respectively) well below the European Union guidance values for fumonisins. Also, the developed method is comparable to commercial lateral flow immunochromatographic assay kits (for toxins in PBS buffer solutions only). However, matrix suppression observed while validating the method makes it unsuitable/impractical for use in the quantification of fumonisin mycotoxins in food samples. This matrix interference observed during the measurement of toxins in maize/corn samples needs to be investigated further. The matrix suppression observed may be because of substances within the samples which inhibit antibody activity, masked fumonisins or non-specific antibody binding activity. In addition, the fact that apparent recoveries in solvent systems tend to overestimate method performance compared to real matrices emphasises that validation solely in buffer systems may provide a misleading impression of robustness. This finding affirms the necessity of validating this approach under realistic food matrix conditions to capture the complexity of interactions and suppression effects. Comparison to the lateral flow immunochromatographic assay also shows that, while both antibody formats are subject to some degree of matrix interference, the lateral flow assay is less variable across maize samples. Nevertheless, the underestimation bias at higher degrees of contamination (>3000 ng/g) detracts from their quantitative reliability. Collectively, the results indicate that both methods, as they currently stand, do not fully meet the requirements of analytical performance criteria required to conduct effective fumonisin monitoring. From a food safety perspective, the implications of the results are profound. Fumonisin concentration underreporting due to poor test performance could result in contaminated maize products reaching the food chain, and subsequently, exposing consumers to unwarranted risk. This has consequences for regulation, since compliance decisions can be made based on low-estimated concentrations of toxins. As the European Committee for Standardisation and EU law observe, such recoveries below acceptable levels (particularly below 90%) must be corrected or at least presented transparently with respect to measurement uncertainty. The resulting IAC strategy, with recoveries usually far below this figure, indicates an urgent need for optimisation before it can be considered appropriate for regulatory or routine monitoring purposes. Although a promising quick method for toxin detection, more research is needed for optimisation of the developed immobilised immuno-affinity column, binding capacity and elution of the toxin afterwards. Research can focus on the optimisation of coupled antibody chemistry, exploring alternative biosupport materials, and evaluation of milder but effective eluting solvents. Complementary analytical methods such as time-of-flight mass spectrometry (ToF-MS) may also be employed in the detection of co-eluting or suppressive matrix components that affect the antibody binding efficiency. Another research area would be in the quantitative determination of the total fumonisins (free and masked) to establish the effect of the masked form of the fumonisins on the functionality of the immuno-affinity purification column. Lastly, developing a robust analytical method that can efficiently detect free and masked fumonisins in complex food matrices will be vital to ensuring public health protection and regulatory compliance.

## 4. Materials and Methods

### 4.1. Analytical Standards, Samples and Chemicals

Analytical standards of fumonisins B1, B2 and B3 (FB1, FB2, FB3) were supplied by Trilogy Analytical laboratories (Washington, DC, USA) with a declared purity of ≥97%. These stock solutions have a certified concentration of 500 µg/mL (FB1), 100 µg/mL (FB2) and 100 µg/mL (FB3) in acetonitrile:water (1:1, *v*/*v*) and was diluted with the same solvents to obtain working standard concentrations of 1 µg/mL. All working standards and stock solutions were stored in amber glass vials at −20 °C. Sodium Sulphate (Na_2_SO_4_) ≥ 99% purity utilised for coupling of antibodies was purchased from Sigma–Aldrich (St. Louis, MO, USA). Ultrapure water (10.2 MΩ) was produced by a Milli-Q integral water purification system (Merck Millipore, Darmstadt, Germany) equipped with an LC-Pak polisher. LC-MS grade acetonitrile (99.99% purity) was purchased from Macron chemicals (Center Valley, PA, USA) and methanol (99.99% purity) was supplied by Fisher Scientific (Leicestershire, UK). Formic acid (98% purity) was supplied by Fluka (Sigma–Aldrich, MO, USA). Solutions of phosphate buffered saline (PBS) were prepared by dissolving commercial PBS granules purchased from Thermo Scientific (Rockford, IL, USA) in ultrapure water. Millex PTFE filters (4 mm, 0.20 µm pore size) for sample filtration was purchased from Merck Millipore (Darmstadt, Germany). Samples consisting of ground homogenised corn naturally contaminated with fumonisins were supplied by Triology Analytical laboratories (Washington, DC, USA). To produce immuno-affinity purification, 5D2 mouse monoclonal IgG1 Antibodies (concentrations of 1.48 µg/mL) were supplied by Neogen Corporation (Irvine, Scotland, UK). Ultralink Biosupports (Product no. 53110) for the coupling of the antibodies were purchased from Thermo Scientific (Waltham, MA, USA).

### 4.2. UHPLC-MS/MS Parameters

For the analysis, an Acquity Ultra High-Performance Liquid Chromatography (UHPLC) system coupled to a Quattro Premier XE Triple Quadrupole mass spectrometer (both Waters Corporation, Milford, CT, USA) was used. Chromatographic separation was undertaken at 40 °C with a flow rate of 400 µL min^−1^ using an Acquity UPLC BEH C18 (2.1 × 50 mm, 1.7 µm) column (Waters Corporation, Milford, CT, USA) with an injection volume of 5 µL. Before sample injection into the UHPLC system, the injection needle was flushed with acetonitrile/water (50:50, *v*/*v*). The mobile phase, adapted from Liu et al. [76], with modifications for enhanced separation of analytes, consisted of (A) water with 0.1% formic acid and (B) methanol with 0.1% formic acid. The method had a total run time of 8 min: after an initial hold time of 0.50 min at 90% A, 90% B was reached after 7 min, followed by a hold time of 0.50 min at 90% B and a final 0.5-min re-equilibration at 90% A. The retention times obtained were 4.99 min (FB1), 5.39 min (FB3) and 5.66 min (FB2).

For mass spectrometric detection, parent and daughter ion selection and the optimisation of cone and collision energies were performed by a direct flow injection of fumonisin standard solutions in water/acetonitrile (1:1, *v*/*v*) into the mass spectrometer using TargetLynx method editor software (TargetLynx XS). Also, MassLynx data acquisition and management software (MassLynx 4.1) was used in the control of the LC-MS/MS instrument. The mass spectrometer was operated in a positive electrospray ionisation (ESI+) mode with source settings as follows: capillary voltage, 3000 V; cone voltage, 40 V; extractor voltage, 3 V; source temperature, 150 °C; desolvation temperature, 400 °C; desolvation gas flow, 500 L/hr; cone gas flow, 50 L/hr. Dynamic multiple reaction monitoring (DMRM) acquisition mode was utilised, thus allowing for the measurement of selected ion transitions within specified retention windows, resulting in a maximisation of the dwell time for each fumonisin analogue. The MRM parameters for fumonisin detection are presented in Table 7. For FB1, FB2 and FB3, two mass transitions were monitored (4.0 identification points) to ensure unambiguous identification and quantification, which is consistent with current LC-MS/MS validation practices reported for mycotoxin analysis in feed and food matrices [77] and the Commission Regulation (EU) 2023/915 [11].

### 4.3. Preparation of Immuno-Affinity Purification Column

To ensure high specificity towards fumonisin B analogues, coupling of the anti-fumonisin mouse monoclonal IgG1 antibody to the UltraLink Biosupport was undertaken according to the manufacturers (Thermo Scientific, Waltham, USA) instructions. A lyotropic salt (0.7M Na_2_SO_4_) was added into the coupling buffer (PBS) to increase coupling efficiency. The coupling was performed at a ratio of 2 mg of UltraLink Biosupport beads per ml of antibody using varying amounts of coupling buffer, under gentle end-over-end mixing for 2 hr at room temperature. The quench solution utilised was ethanolamine ≥ 99.5% purity, purchased from Aldrich Chemicals (St. Louis, MO, USA). The amount of antibody ligand that did not couple to the biosupport beads was measured using a Pierce BCA (bicinchoninic acid) protein assay kit purchased from Thermo Scientific (Waltham, USA) according to the manufacturer’s instructions. Absorbance was measured using a Jenway 6305 spectrophotometer (Bibby Scientific, Staffordshire, UK) at 562 nm. After coupling, the prepared immune-affinity columns were washed with PBS containing 20% ethanol and stored at 4 °C to maintain antibody stability until needed.

### 4.4. Sample Extraction and Immuno-Affinity Clean-Up Procedures

A one step immuno-affinity purification method was developed based on previously established protocols for fumonisin extraction and clean-up from complex food matrices adapted with modifications from Nielsen et al. [78]. Briefly, 1.00 ± 0.01 g samples in 10 mL polypropylene tubes were extracted using 5 mL of 2% formic acid in water using a multi-tube vortexer (VWR, Radnor, PA, USA) for 30 min at room temperature (2500 rpm). Extracts were subsequently centrifuged for 3 min at 4500 rpm (Sorvall Legend centrifuge, Thermo Scientific, Waltham, USA). Resultant aliquots of the supernatant were transferred into 5 mL Eppendorf tubes for further purification and analysis.

For purification, the immuno-affinity column was first equilibrated by flushing with 2 mL of PBS. A 1 mL portion of the raw extract was diluted with 4 mL of PBS buffer solution (1:5 dilution ratio), which was empirically optimised to minimise matrix effects and enhance antibody–antigen binding efficiency on the column. This dilution factor was applied uniformly to all samples to ensure consistency and prevent column overloading. The entire diluted mixture was passed through the immuno-affinity purification column at a flow rate of approximately 1 drop per second, after which the eluent was discarded. The column was then flushed with 5 mL of PBS buffer and blown out with air. The fumonisin mycotoxins are eluted from the column by use of 1 mL MeOH:PBS (1:1, *v*/*v*), which is consistent with recent studies employing mixed organic–aqueous solvents for efficient mycotoxin recovery [79,80]. The eluent is collected and filtered through a 0.20 µm PTFE membrane filter (Merck, Darmstadt, Germany) prior to analysis. The column is finally flushed with 5 mL PBS to remove all organic solvents and equilibrated with PBS for reuse.

### 4.5. Recovery and Matrix Effect Study

The recovery efficiency of the immuno-affinity purification column was investigated by passing solutions of PBS spiked with three concentrations of the toxins (50, 80 and 150 ng/mL) in triplicates through the column. This protocol is consistent with previous reports demonstrating the advantage of aqueous buffers such as PBS in the recovery of polar toxins, as it balances the efficient extraction of the analyte from complex food matrices with compatibility for antibody binding in the column [23,81]. Matrix effect on the performance of the column was determined by spiking certified blank corn samples with the appropriate volume of toxin standards (250 µL, 400 µL and 750 µL of 1000 ng/mL toxin solutions) to yield a final extract of 50 ng/mL, 80 ng/mL and 150 ng/mL of the toxins. Spiked samples were left to stand for 30 min at room temperature to enable the evaporation of solvents and also allow for equilibration between the analytes and sample matrix before commencing with the extraction and clean-up protocols. Also, PBS buffer solutions were spiked with corresponding toxin concentrations. Intra-day repeatability/validation of the method was investigated by measuring these three spiked matrix samples and spiked PBS buffer solutions within a single day.

### 4.6. Lateral Flow Immunochromatographic Assay

The developed method was compared to a single-step commercial Reveal Q+ lateral flow immunochromatographic assay (LFIA) for fumonisin (Neogen Corporation, Lansing, MI, USA) using an AccuScan Pro reader (Neogen Corporation, MI, USA). The assay was performed according to the manufacturer’s instruction. Previous studies have reported the wide application of LFIAs for mycotoxin detection because they are rapid, simple, inexpensive, can be used for on-site screening and have short turnaround time compared to chromatographic methods [82,83]. Briefly, 10 g of well homogenised grounded sample was extracted with 65% methanol and vortexed for 3 min. The sample was allowed to settle and then filtered through a 0.20 µm PTFE membrane filter. 100 µL of the sample was mixed with 200 µL of Neogen sample diluent, and 100 µL of this diluted extract was subsequently transferred into a sample cup. Following this, the Reveal Q+ fumonisin test strip was dipped into the sample cup and the sample was allowed to wick up the test strip for 6 min. Test strips were read within 1 min.

### 4.7. Analysis for Masked and Hydrolysed Fumonisins

Alkaline hydrolysis of corn samples was undertaken to hydrolyse the masked fumonisins according to the method of de Matos et al. [84], with slight modifications. Briefly, 5 g of ground maize sample was mixed with 50 mL of 2M KOH and stirred with a magnetic mixer for 50 min. Next, 50 mL of acetonitrile was added into the mixture and stirred for a further 10 min. This was followed by centrifugation at a speed of 3500 rpm for 15 min in a Sorvall Legend centrifuge (Thermo Scientific, Waltham, USA). After centrifugation, 2 mL of the upper acetonitrile layer was collected and evaporated to dryness under a gentle stream of nitrogen. The obtained residue was redissolved in MeOH:Water (70:30, *v*/*v*) and filtered through a millex 0.22 µL PTFE membrane filter.

## Figures and Tables

**Table 1:** Efficiency of different elution solvents.

Elution Solvent	Expected Toxin Concentration	FB1 Recovery ng/g (%)	FB2 Recovery ng/g (%)	FB3 Recovery ng/g (%)
100% MeOH	50 ng/g	61.2 (122.4%)	74.2 (148.4%)	78.2 (156.4%)
	80 ng/g	79.0 (98.8%)	103.7 (129.6%)	107.7 (134.6%)
0.1M Glycine	50 ng/g	7.7 (15.4%)	4.0 (8%)	6.5 (13%)
	80 ng/g	6.3 (7.9%)	4.7 (5.9%)	5.1 (6.4%)
50% MeOH	50 ng/g	32.0 (64%)	29.4 (58.8%)	35.2 (70.4%)
	80 ng/g	43.0 (53.8%)	51.5 (64.4%)	52.5 (65.6%)

**Table 2:** Intra-day repeatability on recovery of developed method (*n* = 3).

Toxin Source	Spiked Concentration (ng/g)	Recovery (% ± SD)		
		FB1	FB2	FB3
Spiked corn matrix	50	33.8 ± 9%	36.3 ± 7%	35.4 ± 7.2%
	80	27.6 ± 1.8%	31.3 ± 2.3%	30.4 ± 1.7%
	150	28.4 ± 4.8%	31 ± 4%	30.9 ± 3.4%
**MEAN**		**29.9%**	**32.9%**	**32.2%**
Spiked toxins in PBS	50	61.6 ± 0.4%	66.3 ± 3.8%	69.1 ± 4.1%
	80	65.1 ± 8.15%	73.1 ± 6.4%	72.3 ± 6.1%
	150	60.2 ± 2.1%	65.9 ± 2.4%	63.5 ± 2.8%
**MEAN**		**62.3%**	**68.4%**	**68.3%**

**Table 3:** Inter-day reproducibility on recovery of the developed method (*n* = 2).

Toxin Source	Spiked Concentration (ng/g)	Recovery (% ± SD)		
		FB1	FB2	FB3
Spiked corn matrix	50	34.4 ± 1.1%	36.2 ± 0.3%	36.7 ± 2.6%
	80	28.6 ± 1.9%	31.4 ± 0.1%	31.6 ± 2.3%
	150	29 ± 1.1%	31.5 ± 1%	31.5 ± 1.2%
**MEAN**		**30.7%**	**33%**	**33.3%**
Spiked toxins in PBS	50	64 ± 4.7%	69.7 ± 9.7%	70.3 ± 2.4%
	80	67.3 ± 4.4%	74.7 ± 3.2%	74.6 ± 4.5%
	150	62.3 ± 4.2%	66.7 ± 1.6%	66.4 ± 5.7%
**MEAN**		**64.5%**	**70.4%**	**70.4%**

**Table 4:** Recovery of fumonisins by the lateral flow device *n* = 2.

Toxin Source	Spiked Concentration (ng/g)	Total Fumonisins (ng/g)	% Recovery
Spiked corn matrix	400	<300	
	500	338	67.6%
	750	516	68.8%
**MEAN**			**68.2%**
Spiked toxins in PBS	400	317	79.3%
	500	392	78.4%
	750	578	77.1%
**MEAN**			**78.3%**

**Table 5:** Comparison of key performance metrics of the developed immuno-affinity purification method with existing analytical approaches for fumonisin determination in maize.

Parameter	Developed IAC–UHPLC–MS/MS Method	Typical LC–MS/MS Methods (Literature)
LOD	2.5 ng/g	≤2.4 ng/g [68]
LOQ	5 ng/g	≤8.2 ng/g [68]
Recovery (Maize matrix)	30–33%	70–120% [69]
Repeatability (RSD, %)	≤13% (PBS)	≤13% [69]
Analysis Time	<6 min per run	10–18 min per run [69]
Strengths	High sensitivity; reusable IAC column; short run time	Widely validated; good matrix robustness
Limitations	Severe matrix suppression; low recovery in maize	Expensive equipment; longer prep time

**Table 6:** Method performance.

Reference Sample (corn)	Expected Certified Conc. (Total Fumonisin) (ng/g)	Lateral Flow Device		Immuno-Affinity Purification	
		Conc. (ng/g)	% Recovery	Conc. (ng/g)	% Recovery
A	0	<300		189	189%
B	700	479	68.4%	403	57.6%
C	1000	638	63.8%	463	46.3%
D	1500	997	66.5%	548	36.5%
E	2200	1433	65.1%	201	9.1%
F	2400	1831	76.3%	632	26.3%
G	3200	1891	59%	556	17.4%
H	3600	2311	64.2%	684	19%
I	4000	2162	54.1%	590	14.8%
J	4700	2666	56.7%	674	14.3%

**Table 7:** MRM parameters for fumonisin detection.

Analyte	Parent Ion (m/z)	Cone Voltage (V)	Daughter Ions (m/z)	Collision Energy (V)	Dwell (S)
FB1	722.25	50	334.45	40	0.125
			352.45	35	0.125
FB2	706.30	55	318.45	40	0.125
			336.50	35	0.125
FB3	706.35	55	336.50	35	0.125
			354.50	30	0.125

## Data Availability

The original contributions presented in this study are included in the article. Further inquiries can be directed to the corresponding author.
